# Expression of Concern: *Characterization of TFIIE-regulated genes by transcriptome analysis*

**DOI:** 10.55730/1300-0152.2750

**Published:** 2025-06-01

*Published in Turkish Journal of Biology, Volume 48, Issue 6, Pages* 442 *–* 455, *https://doi.org/10.55730/1300-0152.2718*

The Editors of *Turkish Journal of Biology* wish to issue an Expression of Concern regarding the article entitled:

**“*Characterization of TFIIE-regulated genes by transcriptome analysis*”** by Serdar Baysal published on 20.12.2024

Following the publication of this article, concerns have been raised regarding

- Potential violations of academic ethical standards in the published study
- Potential intellectual property infringement, including plagiarism
- An academic sanction reportedly imposed on the author by the affiliated university following an institutional disciplinary investigation

An investigation is currently underway to assess the validity of these concerns. In accordance with the guidelines of the Committee on Publication Ethics (COPE), and to maintain transparency with our readership, we are issuing this Expression of Concern while the matter is being reviewed.

This notice does not imply conclusive evidence of misconduct or error at this stage, but serves to alert readers to potential issues affecting the reliability of the article. Further editorial action will be taken as appropriate once the investigation is complete.

We will update this notice in due course based on the outcome of the investigation.

